# About Oxiphosphate

**Published:** 1893-05

**Authors:** 


ARTICLE VI.

### ABOUT OXIPHOSPHATE.

The fluid of the oxiphosphate sometimes crystallizes. This only shows it is a little too rich. Add a few drops of water and these crystals will soon disappear. Sometimes it is only necessary to heat the bottle in hot water. If water is added care must be used to add only enough to reduce it to a syrupy condition; a single drop more than this will spoil it. If the mix crumbles it generally shows that the fluid is too thin; it can be evaporated to a syrup if great care is used. Sometimes this crumbling is the fault of the powder; either it was not properly manufactured, or has been exposed to moisture.

A smooth, creamy cement will be more easily produced if the fluid and powder are put on the slab separately, and mixed by drawing the powder gradually into the fluid, stirring with a narrow spatula. If the slab is cool the cement will set the more slowly; if it is mixed on a quite warm slab, it is more apt to crumble; if moderately cool, it should set so quickly that the tooth must be protected by the rubber-dam, and the cavity thoroughly dried before the cement is mixed, and it must be used before it begins to set. Under these conditions, if it is mixed soft, it will adhere to the walls of the cavity and make a much more durable filling.

The cavity should be so nicely and completely filled, while the cement is still soft, that it will not require any smoothing, or rubbing, or cutting, or scraping, or even reducing with sandpaper. It must be brought to a perfect form and finished while soft, so that it will harden with a gloss. It will then have a harder and more enduring surface. Pressing it while hardening makes it crumble, for you break the forming crystallization.

The rubber-dam should remain on till the cement becomes pretty hard, and then, before it gets wet, it should be protected with paraphine. This must be carried to the surface on a heated spatula, so that the paraphine will be so hot as to flow smoothly in a thin layer all over, immediately it touches the surface.

Is all this too much trouble? It should never be too much trouble to do our best in everything.

There are two ways of making large oxiphosphate fillings permanent, more permanent and better than large amalgam or gold filling. One is to almost fill the cavity with the oxiphosphate, and then, while it is soft, press into and overlay its surface with small pieces of soft alloy or of crystal gold. After a few minutes, when the cement is sufficiently hard, finish the filling by adding a little more metal and condense. If the cement is allowed to harden first a small portion has to be cut away, then the metal added, the first part being worked into pits or grooves made in the cement.

Another way is to cut and bend a thin piece of gold plate to fit the surface of the cavity. The pattern can be obtained by pressing on the surface a piece of lead or tin foil. A pin from an old tooth, or a strip of the gold bent V shape can be soldered on the under side. When the cavity is quite filled with cement, and still soft, press the plate on to it so that the pin or V attachment will sink into the cement, and the plate be on a level with the surface of the tooth.

The advantage of a filling of oxiphosphate covered in either of these ways is that by the cement sticking to the walls it makes a perfect filling, and thus protected the cement will not deteriorate.

The following way of making an artificial crown is not new, but some may be benefited by a description.

Make a gold band to fit over the root and pass well down under the gum, and up to nearly articulate with the opposing tooth. Solder a cap on this so that it shall represent the grinding surface of the tooth. Partly fill this cup and press it thoroughly to place. A bite on it with a piece of "tea lead" foil on top will show when it properly articulates. The tea lead is placed on the grinding surface, so that when it is removed the articulation will not be quite close. Of course such a crown will not do for a front tooth.—*Items of Interest.*

